# Supplementary material for: Health and economic burden of foodborne zoonotic diseases in Amhara region, Ethiopia
Source: PLoS One. 2021 Dec 31;16(12):e0262032. doi: 10.1371/journal.pone.0262032 (PMC8719781; doi:10.1371/journal.pone.0262032)
Supplement: S1 Questionnaire — (DOC) [file pone.0262032.s001.doc]

**Questionnaire on health and economic burden of foodborne zoonotic diseases**

**Health and economic burden of foodborne zoonotic diseases in Amhara region, Ethiopia**

S.A. Mekonnen, A. Gezehagn, A. Berju, B. Haile, H. Dejene, S. Nigatu, W. Molla and W. T. Jemberu

Date____________

Questionnaire number__________

Town _____________

1. Name of interviewee _________________
2. Gender: Male Female
3. Age ___________________
4. Level of education______________
5. Do you have family? Yes No
6. How much is the estimate of your family income per month? ___________________
7. Do you know about zoonotic-diseases? Yes No
8. Family size ___________
9. Tell me name of zoonotic diseases you know.

______________________________________________________________________

1. Do you know whether zoonotic-diseases can be acquired from food?

Yes No

1. Did you experience zoonotic-diseases in your family in the last two years?

Yes No

If yes, how many of your family experienced zoonotic-diseases in the last two years? _______

1. What were the clinical signs observed when you or your family had got the zoonosis?

______________________________________________________________________

1. Have you encounter a disease with sign of diarrhoea in your family in the last two years? Yes No

If yes, how many of your family experienced disease with sign of diarrhoea? _________

1. Do you and your family sick treatment when affected by a disease with sign of diarrhoea?

Yes No

1. How many of your family members affected by a disease with sign of diarrhoea in the last **two year**s were treated? ______ How many were not treated? _______
2. Do you treat disease with sign of diarrhoea? Yes No
3. How long you and your family stay before seeking health service? ____________
4. Where do you and your family get treatment service to disease with sign of diarrhoea?
5. By yourself b. Other traditional healer c. Holy water d. Clinic/Hospital

|  |  | | How much do you receive/pay per patient | How long it takes to go to the site, get holy water come back home? |
| --- | --- | --- | --- | --- |
| 1 | By yourself | |  |  |
| 2 | Traditional healer | |  |  |
| 3 | Holy water | |  |  |
| 4 | Clinic/Ho | |  |  |
|  | |  | | |

1. In clinic/Hospital, in average:
2. How much do you pay for the diagnosis? ________________________
3. How long did it take to finish diagnosis i.e. before starting the treatment? _________________
4. Time taken from the beginning to the end of the treatment?____________ days
5. How long a patient follow treatment being inpatient (if any) ____________
6. How long a patient follow the treatment being out patient (if any) ____________
7. How long sick leave was given (if available)____________ days
8. Does a sick person with the sign of diarrhoea need care? Yes No

If yes, how long it take in hours per day to care a person with sign of diarrhoea? _______

1. How long it takes to cure from the disease with sign of diarrhoea after the treatment? ___________________.
2. How much is the average income of a person who give care for a sick person per hour for labor? _____________________
3. What are the costs of the drug used to treat a person with sign of diarrhoea in a clinic/hospital? ________________ ETB.
4. Does a person who gets sick with a disease with sign of diarrhoea continue working while he/she is sick? Yes No
5. If stop working, for how long stop working? _____________ days
6. Have you ever encountered a disease with sign of diarrhoea? Yes No

If yes, does the disease have any discomfort? Yes No

1. How long did you feel any discomfort associated with the disease with sign of diarrhoea? ____________________
2. Does anyone from your family died after developing a disease with sign of diarrhoea?

Yes No

1. At what age was he/she died? _____________________
2. How long he stayed showing clinical signs before death? _____________________
3. At what of age the person died ? __________
4. Does your family take preventive measures against diseases producing diarrhoea?

Yes No

1. List the measures your family practice to prevent diseases producing diarrhea.

____________________________________________________________________________________________________________________________________________________

1. Estimate the measures your family practice to prevent diseases producing diarrhea in costs.

__________________________________

**Retrospective Data collecting format**

Data will be collected from two health centers in each of the four towns: Debark, Gondar, Bahir Dar and Lalibela. Cases with signs of diarrhoea in the last two years will be selected from patient records from two health centers in each of the four towns. Then the following data will be collected from the record.

1. Name of the patient
2. Gender: Male Female
3. Age ___________________
4. What were the lists of differential diagnoses? ________________________________

____________________________________________________________________

1. What was the tentative diagnosis? ________________
2. Was there any laboratory technique made to confirm the problem? Yes No

If yes, what was the final diagnosis made? _______________________
