# Supplementary material for: Health and economic burden of foodborne zoonotic diseases in Amhara region, Ethiopia
Source: PLoS One. 2021 Dec 31;16(12):e0262032. doi: 10.1371/journal.pone.0262032 (PMC8719781; doi:10.1371/journal.pone.0262032)
Supplement: Attachment — Submitted filename: PONE-D-21-22519_reviewer.pdf [file pone.0262032.s003.pdf]

PLOS ONE

Health and economic burden of foodborne zoonotic diseases in Amhara region, Ethiopia

--Manuscript Draft--

**Manuscript Number:**	PONE-D-21-22519
**Article Type:**	Research Article
**Full Title:**	Health and economic burden of foodborne zoonotic diseases in Amhara region, Ethiopia
**Short Title:**	Health and economic burden of foodborne zoonotic diseases
**Corresponding Author:**	Sefinew Alemu Mekonnen, PhD<br>University of Gondar Faculty of Veterinary Medicine<br>Gondar, Amhara region ETHIOPIA
**Keywords:**	Amhara Region, Ethiopia, Economic burden, Foodborne, Health burden, Zoonotic diseases
**Abstract:**	Diseases from food of animal origin are common health problems in Ethiopia. This study was carried out to estimate health and economic burden, and to identify demographic factors associated with community awareness of foodborne zoonotic diseases in Amhara region, Ethiopia. Data was collected from 435 households in three towns: Gondar, Lalibela and Debark, proportional to the households in the towns. A retrospective data was also collected from health records in each town. The health burden due to zoonotic diseases was estimated at 0.2, 0.1 and 1.3 DALYs per household per year and at 73.2, 146.6 and 1,689.5 DALYs out of 100,000 populations per year in Gondar, Lalibela and Debark, respectively. The overall health burden due to foodborne zoonotic diseases (aggregated over the 435 households in the three towns) was estimated to be 89.9 DALYs per 100,000 populations per year. The economic impact of foodborne zoonotic diseases in the three towns of Amhara regional state was 278.98 Ethiopian Birr (ETB) (1ETB = 0.025 US Dollar) per household per year and 121,355.68 ETB per year in the three towns. Costs of preventive measures followed by costs of patients' time made the highest contribution while costs of diagnosis made the lowest contribution to the total economic burden of foodborne zoonotic diseases. From a total of 435 respondents, 305 (70.1%) had known the presence of zoonotic diseases. Level of education, number of families in the house and income were highly associated with awareness of zoonosis. Although majority of respondents had known zoonotic diseases exists (70.1%) and disease can be acquired from animal source food (63.2%), the health and economic burden associated to foodborne zoonotic diseases are still high. Therefore, changing mindset and practical training aiming in controlling foodborne zoonotic diseases is suggested to the community in the health improvement extension service.
**Order of Authors:**	Sefinew Alemu Mekonnen
	Agegnehu Gezahegn
	Adugna Berju
	Belete Haile
	Haileyesus Dejene
	Seleshe Nigatu
	Wassie Molla
	Wudu Temesgen Jemberu
**Additional Information:**	
**Question**	**Response**
**Financial Disclosure**	AB, BH, HD, SAM.The funders had no role in study design, data collection and analysis, decision to publish, or preparation of the manuscript.

Powered by Editorial Manager® and ProduXion Manager® from Aries Systems Corporation

Enter a financial disclosure statement that describes the sources of funding for the work included in this submission. Review the submission guidelines for detailed requirements. View published research articles from *PLOS ONE* for specific examples.<br><br>This statement is required for submission and **will appear in the published article** if the submission is accepted. Please make sure it is accurate.<br><br>**Unfunded studies**<br>Enter: *The author(s) received no specific funding for this work.*<br><br>**Funded studies**<br>Enter a statement with the following details:<br>• Initials of the authors who received each award<br>• Grant numbers awarded to each author<br>• The full name of each funder<br>• URL of each funder website<br>• Did the sponsors or funders play any role in the study design, data collection and analysis, decision to publish, or preparation of the manuscript?<br>• **NO** - Include this sentence at the end of your statement: *The funders had no role in study design, data collection and analysis, decision to publish, or preparation of the manuscript.*<br>• **YES** - Specify the role(s) played.<br><br>* typeset	
**Competing Interests**<br><br>Use the instructions below to enter a competing interest statement for this submission. On behalf of all authors, disclose any competing interests that could be perceived to bias this work—acknowledging all financial support and any other relevant financial or non-financial competing interests.<br><br>This statement is required for submission and **will appear in the published article** if the submission is accepted. Please make	The authors have declared that no competing interests exist.

Powered by Editorial Manager® and ProduXion Manager® from Aries Systems Corporation

sure it is accurate and that any funding sources listed in your Funding Information later in the submission form are also declared in your Financial Disclosure statement.<br><br>View published research articles from *PLOS ONE* for specific examples.<br><br>**NO authors have competing interests**<br><br>Enter: *The authors have declared that no competing interests exist.*<br><br>**Authors with competing interests**<br><br>Enter competing interest details beginning with this statement:<br><br>*I have read the journal's policy and the authors of this manuscript have the following competing interests: [insert competing interests here]*<br><br>* typeset	
**Ethics Statement**<br><br>Enter an ethics statement for this submission. This statement is required if the study involved:<br><br>• Human participants<br>• Human specimens or tissue<br>• Vertebrate animals or cephalopods<br>• Vertebrate embryos or tissues<br>• Field research<br><br>Write "N/A" if the submission does not require an ethics statement.<br><br>General guidance is provided below. Consult the submission guidelines for detailed instructions. **Make sure that all information entered here is included in the Methods section of the manuscript.**	For the questionnaire-based study, no formal approval by the Institutional Review Board of the University of Gondar was required. Participating farmers were instructed about the content and goal of the questionnaire before the start of the interview. All data were analyzed and reported anonymously. The data was collected by the first author.

**Format for specific study types**<br><br>**Human Subject Research (involving human participants and/or tissue)**<br>• Give the name of the institutional review board or ethics committee that approved the study<br>• Include the approval number and/or a statement indicating approval of this research<br>• Indicate the form of consent obtained (written/oral) or the reason that consent was not obtained (e.g. the data were analyzed anonymously)<br><br>**Animal Research (involving vertebrate animals, embryos or tissues)**<br>• Provide the name of the Institutional Animal Care and Use Committee (IACUC) or other relevant ethics board that reviewed the study protocol, and indicate whether they approved this research or granted a formal waiver of ethical approval<br>• Include an approval number if one was obtained<br>• If the study involved *non-human primates*, add *additional details* about animal welfare and steps taken to ameliorate suffering<br>• If anesthesia, euthanasia, or any kind of animal sacrifice is part of the study, include briefly which substances and/or methods were applied<br><br>**Field Research**<br><br>Include the following details if this study involves the collection of plant, animal, or other materials from a natural setting:<br>• Field permit number<br>• Name of the institution or relevant body that granted permission	
**Data Availability**<br><br>Authors are required to make all data underlying the findings described fully available, without restriction, and from the time of publication. PLOS allows rare exceptions to address legal and ethical concerns. See the PLOS Data Policy and FAQ for detailed information.	Yes - all data are fully available without restriction

A Data Availability Statement describing where the data can be found is required at submission. Your answers to this question constitute the Data Availability Statement and **will be published in the article**, if accepted.<br><br>**Important:** Stating 'data available on request from the author' is not sufficient. If your data are only available upon request, select 'No' for the first question and explain your exceptional situation in the text box.<br><br>Do the authors confirm that all data underlying the findings described in their manuscript are fully available without restriction?	
**Describe where the data may be found in full sentences. If you are copying our sample text, replace any instances of XXX with the appropriate details.**<br><br>• If the data are **held or will be held in a public repository**, include URLs, accession numbers or DOIs. If this information will only be available after acceptance, indicate this by ticking the box below. For example: *All XXX files are available from the XXX database (accession number(s) XXX, XXX.).*<br>• If the data are all contained **within the manuscript and/or Supporting Information files**, enter the following: *All relevant data are within the manuscript and its Supporting Information files.*<br>• If neither of these applies but you are able to provide **details of access elsewhere**, with or without limitations, please do so. For example:<br><br>*Data cannot be shared publicly because of [XXX]. Data are available from the XXX Institutional Data Access / Ethics Committee (contact via XXX) for researchers who meet the criteria for access to confidential data.*<br><br>*The data underlying the results presented in the study are available from (include the name of the third party*	All relevant data are within the manuscript and its Supporting Information files.

and contact information or URL).<br>• This text is appropriate if the data are owned by a third party and authors do not have permission to share the data.<br><br>* typeset	
Additional data availability information:	Tick here if your circumstances are not covered by the questions above and you need the journal's help to make your data available.

Powered by Editorial Manager® and ProduXion Manager® from Aries Systems Corporation

Manuscript
Click here to access/download;Manuscript;Mekonnen.docx

Health and economic burden of foodborne zoonotic diseases in Amhara region, Ethiopia

S.A. Mekonnen[1*], A. Gezehagn[2], A. Berju[1], B. Haile[1], H. Dejene[1], S. Nigatu[1], W. Molla[1] and W. T. Jemberu[1]

[1]Department of Veterinary Epidemiology and Public Health, College of Veterinary Medicine and Animal Sciences, University of Gondar, P.O. Box 196, Gondar, Ethiopia.

[2]Centeral Gondar zone Animal Quarantine Center, Gondar, Ethiopia.

* Corresponding author.

E-mail address: asefinew@gmail.com

Abstract

Diseases from food of animal origin are common health problems in Ethiopia. This study was carried out to estimate health and economic burden, and to identify demographic factors associated with community awareness of foodborne zoonotic diseases in Amhara region, Ethiopia. Data was collected from 435 households in three towns: Gondar, Lalibela and Debark, proportional to the households in the towns. A retrospective data was also collected from health records in each town. The health burden due to zoonotic diseases was estimated at 0.2, 0.1 and 1.3 DALYs per household per year and at 73.2, 146.6 and 1,689.5 DALYs out of 100,000 populations per year in Gondar, Lalibela and Debark, respectively. The overall health burden due to foodborne zoonotic diseases (aggregated over the 435 households in the three towns) was estimated to be 89.9 DALYs per 100,000 populations per year. The economic impact of foodborne zoonotic diseases in the three towns of Amhara regional state was 278.98 Ethiopian

Birr (ETB) (1ETB = 0.025 US Dollar) per household per year and 121,355.68 ETB per year in the three towns. Costs of preventive measures followed by costs of patients' time made the highest contribution while costs of diagnosis made the lowest contribution to the total economic burden of foodborne zoonotic diseases. From a total of 435 respondents, 305 (70.1%) had known the presence of zoonotic diseases. Level of education, number of families in the house and income were highly associated with awareness of zoonosis. Although majority of respondents had known zoonotic diseases exists (70.1%) and disease can be acquired from animal source food (63.2%), the health and economic burden associated to foodborne zoonotic diseases are still high. Therefore, changing mindset and practical training aiming in controlling foodborne zoonotic diseases is suggested to the community in the health improvement extension service.

Keywords: Amhara Region, Ethiopia, Economic burden, Foodborne, Health burden, Zoonotic diseases.

Introduction

Zoonotic diseases are diseases transmissible between people and animals. Nearly two-thirds of human infectious diseases exerting heavy public health and economic burden to the global community originate from animals [1, 2]. In the absence of proper care of the relation between animal and human populations, zoonotic diseases can lead to a serious risk of public health with huge economic consequences [3]. Zoonotic diseases threaten the health and productivity of animals, the livelihood of people, and cause illness and death in consumers [4]. Worldwide, the direct cost of zoonotic diseases over the last decade has been estimated to be more than $20

billion with over $200 billion indirect losses [5]. Zoonotic diseases and diseases recently emerged from animals have been estimated to contribute more than a quarter of the disability-adjusted life years (DALYs) lost to infectious diseases in low income countries such as sub-Saharan Africa [6]. In these countries, however, the economic and health burden associated to zoonotic disease is often given low attention and is largely unknown [4]. In order to obtain a better understanding of their health impact, calculating summary measures of population health such as the DALY is necessary. The attention given to zoonotic diseases has however focused more on emerging zoonotic diseases that pose global economic and health threats than endemic zoonotic diseases [7-9]. Quantitative data on health and economic burden of zoonotic diseases may contribute in changing mindset to make informed decision on prevention and control of endemic zoonotic diseases.

Foodborne diseases have enormous impacts on the health and livelihoods of people around the globe and are of great concern to consumers, producers, and policymakers. Although many articles have been published on (foodborne) zoonotic diseases worldwide [10-14]), specific information on health and economic impact of (foodborne) zoonotic diseases in developing countries is limited [4]. So in order to get a better understanding of the impacts of zoonotic diseases specifically for the situation in developing countries, estimates of the health and economic burden of foodborne zoonotic diseases are needed. By estimating the burden of foodborne zoonotic diseases, awareness of the economic and health consequences of these diseases can be created, which is a first step for prevention and an important part of motivating policy makers to take preventive measures against foodborne zoonotic diseases. Particularly, the

estimation of the burden of foodborne zoonotic diseases in households can be helpful, as it provides households with estimate to assess their own situation.

Ethiopia has the second largest human population in Africa and the largest in livestock population in the continent [15]. About 80% of Ethiopians are dependent on agriculture and have direct contact with livestock or other domestic animals which creates opportunity for infection and spread of zoonotic diseases between humans and animals [4]. This and having a large population of low income livestock farmers, Ethiopia also ranks very high in the health burden of zoonotic diseases [13]. The situation is very much pronounced in rural communities of Ethiopia where animals and humans share the same microenvironment including a shelter [16]. Besides, the handling and widespread habit of raw beef consumption in Ethiopia can favor the spread of foodborne zoonotic infection. Raw meat without appropriate temperature control is available in open-air in local retail shops for sale and minced meat, 'Kitfo' in Ethiopian Amharic language, is served as raw or under cooked at restaurants.

There are a lot of foodborne diseases in Ethiopia [4]. Salmonellosis is one of the most common foodborne bacterial zoonotic diseases identified from food animals in Ethiopia [17]. Non-typhoidial *Salmonella* represents an important human and animal pathogen worldwide [18]. In humans, in addition to concern about foodborne zoonotic diseases caused by *Salmonella* organisms, concern has also been raised about the impact of acquired antimicrobial resistance transferred among these organisms [19]. Listeriosis is an important emerging foodborne bacterial zoonotic infections worldwide. Ready-to-eat food-mediated listeriosis infection in humans has been documented by several workers from different parts of the world [21, 22]. In Ethiopia,

Molla et al. [20] demonstrated widespread occurrence and distribution of *L. monocytogenes* and other *Listeria* species in retail meat and milk products in Addis Ababa. Despite the potential threat to human health posed by listeriosis, there is scarcity of data on the public health and economic burden associated to this disease. From, helminthes, taeniasis caused by *Tinea saginata* is common infection associated to eating raw or undercooked beef in many sections of human population in the country [23].

Relatively “many articles” are published on the epidemiology of foodborne zoonotic diseases in Ethiopia [4, 16, 24-32]. However, to our knowledge, there is no such published data on the health and economic burden of foodborne zoonotic diseases [4]. The present study aimed at estimating the impact of foodborne zoonotic diseases on health and economy, and at identifying factors associated with awareness of the people about these diseases in Amhara region, Ethiopia.

Materials and methods

2.1. Study area

The study was conducted in three towns selected from Amhara regional state: Gondar, Debark and Lalibela. These towns were selected because they are destinations for large number of tourists. The 2014-2017 projected populations for the three towns were 306, 246 for Gondar, 30,781 for Debark and 27, 200 for Lalibela [32].

2.2. Study population and animal source food consumption habits

A total of 435 households obtained from three selected towns: 286 from Gondar, 71 from Lalibela, and 78 from Debark were included in the study. The sample size was allocated proportional to the size of the households for each of the towns. Within each town, respondents were recruited haphazardly.

The studied households consume both cereals and animal products. Overall diets are dominated by grains and that both per capita consumption and food budget shares of animal source food are low. Nowadays, the quantity of animal source food consumption is increasing although the increase is modest because of relatively higher price of animal products. Consumption of animal source food is also seasonal. Orthodox Christians don't consume animal source food during fasting season and on Wednesdays and Fridays except the 50 days running from Easter. Animal source food consumption peak is associated with major religious events [33]. No such seasonality is seen during the Muslims' major fasting season of Ramadan. Both religious groups are dependent on limited types of animals for meats due to cultural taboo for some food animals such as pigs. The per capita consumption in towns is higher than the per capita consumption in rural areas linked to income differences and associated resulting changes in household preferences [34]. A cow or an ox is commonly butchered for the sole purpose of selling and most people buy raw meat from butchers and cook and consume within their home. In special occasions, people have a cultural ceremony of slaughtering cow or ox and sharing among the group. In addition to consuming completely or partly cooked meat, eating raw meat is a common practice. At the larger butcheries, many frequently sit and eat traditional meal of higher quality raw meat [35].

2.3. Design of the questionnaire and data collection

A questionnaire was prepared to collect data on demography, household health expenses, treatment service, time spent on seeking health service, and risks associated to diarrhea disease. Most of the questions were closed questions to make ease of the analysis. The questionnaire was pre-tested by a administering it on in 5 people that have similar characteristics to the study participants outside the study. The questionnaire was prepared in English and translated into local language (Amharic) for administration. The English version of the questionnaire is provided in Supplementary material 1 (S1).

The questionnaire was administered by face-to-face interviews to the 435 respondents recruited from the study towns. In addition a retrospective data was collected from records of health centers serving the study towns. In the retrospective data collection, cases with signs of diarrhea referred for laboratory confirmation in the last two years were sought from patient records of two health centers in each of the three towns. In accordance with the local legislation and institutional requirements, ethical review and approval was not required for such questionnaire study. A verbal informed consent should be obtained from respondents before the start of the interview and the data should be analyzed and reported anonymously.

2.4. Data management and analysis

The health and economic impact of foodborne zoonotic diseases was estimated based on the number of cases of diarrhea obtained from respondents and the proportion of diarrhea case caused by foodborne zoonotic diseases obtained from health center records. The proportion of diarrhea cases caused by foodborne zoonotic diseases (0.49) derived by dividing the number of diarrhea caused due to confirmed foodborne zoonotic diseases (653) to the total number of diarrheic cases confirmed by laboratory (1332) from the last one year case records of three health centers. As respondents were not happy to tell their household size, average household size of 4.8 for the region was used from secondary sources [36]. Data used to estimate the impact of foodborne zoonotic diseases on health and economy and to identify factors associated with awareness of the people about these diseases in Amhara region, Ethiopia are available in Supplementary material 2 (S2 Table).

2.5. Calculation of impacts of foodborne zoonotic diseases on health

Health impact of foodborne zoonotic diseases was estimated by summary disease measure of disability adjusted life years (DALY) which is sum of years of life lost (YLL) due to premature mortality and years lived with disability (YLD) [37, 38].

Years of life lost: Years of life lost due to death associated to foodborne zoonotic diseases was estimated from expected years of life lost at death derived from the standard life table for Ethiopia as given by WHO and from the number of deaths due to foodborne zoonotic diseases as

$$YLL_i = \sum_{i=Birth}^{n}(N_i^D * E_i) \quad (1)$$

Where

YLL_i = Years of life lost due to death associated with foodborne zoonotic disease in household with age *i*

N_i^D = Number of human death in household with age *i*

E_i = Life expectancy of the concerned age category of i

Years lived with disability

Years lived with disability was estimated separately for disability due to the diseases and due to the medication.

Years lived with disability due to diarrhea: Years lived with disability due to foodborne zoonotic disease associated diarrhea was estimated from number of diarrheic patients, average duration of stay with the diarrhea and disability weight of 0.17 for diarrhea, averaged from the disability report for mild, moderate and sever cases of diarrhea from Salomon et al. [39] as

$$YLD_D = N^{PT} * T * DW_D \quad (2)$$

Where:

YLD_D = Years lived with disability due to foodborne zoonotic disease associated diarrhea in household

N^{PT} = Number of diarrheic patients in household

T = Average duration of stay with diarrhea associated to the foodborne zoonotic diseases in household

DW_D = Disability weight of diarrhea

Years lived with disability due to medication: This was estimated from the number of patients treated for foodborne zoonotic diseases associated diarrhea, average duration of treatment and disability weight of medication. Disability of weight of daily medication for generic uncomplicated diseases is 0.07 as estimated by Haagsma et al. [40].

$$YLD_{Mi} = N_i^{PT} * T_i * DW_M \quad (3)$$

Where:

YLD_{Mi} = Years lived with disability due to medication of diarrhea associated with foodborne zoonotic disease in household *i*

N_i^{PT} = Number of patients treated for diarrhea associated with foodborne zoonotic diseases in household *i*

T_i = Average duration of treatment of with diarrhea associated to the foodborne zoonotic diseases in household *i*

DW_M = Disability weight of medication from the literature for generic uncomplicated diseases

Total DALYs in household was the summation of years of life lost due to death, years lived with disability due to the diseases and years lived with disability due to medication of diarrhea associated to foodborne zoonotic diseases as

$$\text{DALY} = \sum_{i=Birth}^{n}(YLL_i) * YLD_D + YLD_M \quad (4)$$

Where:

YLL_i = Years of life lost due to death associated to foodborne zoonotic disease of individual with age *i*

YLD_D = Years lived with disability due to foodborne zoonotic disease associated diarrhea

YLD_M = Years lived with disability due to medication associated to foodborne zoonotic disease associated diarrhea

2.6. Estimating economic impacts of foodborne zoonotic diseases

The economic impact of foodborne zoonotic diseases for study participant households was aggregated from the cost of diagnosis, cost of drug, cost of informal carers and cost of patients' time per year. Economic impact due to illness falls into two broad categories, namely direct and indirect costs. Direct costs are costs incurred for health care services such as costs of diagnosis and costs for drugs. Indirect costs are opportunity costs due to productive working time losses resulting from illness and to other healthy members of household [41].

Costs of diagnosis of foodborne zoonotic diseases

$$C_i^{Di} = N_i^{PT} * P_i^{Di} \quad (5)$$

Where:

C_i^{Di} = Costs of diagnosis of foodborne zoonotic diseases in household *i*

N_i^{PT} = Number of patients treated for foodborne zoonotic disease in household *i*

P_i^{Di} = Price of diagnosis per case in household *i*

Costs of drugs

$$C_i^{Dr} = N_i^{PT} * P_i^{Dr} \quad (6)$$

Where:

C_i^{Dr} = Costs of drug to treat a foodborne zoonotic disease case in household *i*

N_i^{PT} = Number of patients treated for foodborne zoonotic disease in household *i*

P_i^{Dr} = Price of drug per case in household *i*

Costs of informal carers

$$C_i^{IC} = N_i^{PT} * T_i^{IC} * P_i^{Lh} \quad (7)$$

Where:

C_i^{IC} = Costs of informal carers in household *i*

N_i^{PT} = Number of patients treated for foodborne zoonotic disease in household *i*

T_i^{IC} = Time taken by informal carers to nurse a patient in household *i*

P_i^{Lh} = Price of labor (25 Ethiopian Birr/hour) in Amhara region. The official exchange rate during the study year was One ETB for 0.025 USD.

Costs of patient's time

$$C_i^{TP} = ((N_i^{PT} * T_i^{IT}) + (N_i^{PuT} * T_i^{IPuT})) * P_i^{Lh} \quad (8)$$

Where:

C_i^{PT} = Costs of patients' time associated to illness in household *i*

N_i^{PT} = Number of patients treated for foodborne zoonotic disease in household *i*

T_i^{IL} = Productive working time losses resulting from illness and treatment in patients treated in household *i*

N_i^{PuT} = Number of patients untreated for foodborne zoonotic disease in household *i*

T_i^{IPuT} = Productive working time losses resulting from illness in patients untreated in household *i*

P_i^{Lh} = Price of labor (25 Ethiopian Birr/hour) in Amhara region

2.7. Analyzing factors associated with awareness of foodborne zoonotic diseases

Descriptive statistics such as frequency distribution and percentages were used to summarize the data. Factors associated with awareness of foodborne zoonotic diseases were analyzed using logistic regression. The dependent binary variable was awareness of foodborne zoonotic disease (yes or no). Independent variables were study town (Gondar, Lalibela and Debark), gender (male and female), household size (one person or >one person), age in years (<35, >35 to 50 and >50), level of education (illiterate, general education, college education), and income per month (<2500 Ethiopian Birr (ETB) 2501-5000 ETB, 5001-7500 ETB and >7500 ETB.

Independent variables were screened using univariable logistic regression. Pair-wise correlations between independent variables were evaluated using the Spearman Rank correlations for variables significant in the univariable analysis. If two variables had a correlation coefficient of ≥0.7, only one of the variables was included in the multivariable analysis. Variables statistically significant at $P < 0.05$ in the univariable analyses were tested by multivariable model. Variables in multivariable models with $P < 0.05$ from the Wald test were retained. All two-way interactions between variables in the final multivariable models were tested. The final multivariable model was built by backward elimination. Confounding was checked during the model building process by evaluating the change in the coefficients of other variables when a variable was eliminated from the models. If this change in beta estimate was >30%, the variable was considered a

confounder. The analyses were done using Stata statistical software release 14 (Stata Corp LLC, USA).

Results

3.1. Health and economic burden of foodborne zoonotic diseases

The health and economic impact estimates were based on three diarrhea causing foodborne zoonotic diseases: salmonellosis, listeriosis and taeniasis which were confirmed in health centers of the three towns Gondar, Lalibela and Debark in North West Ethiopia. The minimum, average and maximum values of the most important input items for the health and economic impact estimate are summarized in Table 1.

Table 1: Summary of the minimum, average and maximum annual values of parameters used in estimating health and economic burden of foodborne zoonotic diseases (N =435).

Model parameters	Minimum	Average	Maximum
Number of diarrheic patients per household	1	1.5	3
Number of patients treated per household	0	0.9	2
Costs of diagnosis (ETB[1])	5	36.4	350
Costs of treatment and drug (ETB)	20	82	500
Duration of treatment (days)	0	1.7	9
Sick leave time (days)	0	4.2	33
Waiting for diagnosis (hours)	0	3.2	12

Time taken of informal cares (days)	0	0.7	6
Number of dead people per household	0	0.03	1
Age of death of individuals (years)	0	0.08	5
Days elapsed before seeking treatment (Days)	0	2.24	7
Costs of prevention of diarrhea (ETB)	0	431	3,000

[1]Ethiopian Birr

An average annual human mortality rate of 0.01 was recorded associated to foodborne zoonotic diseases. The minimum, average and maximum income of respondents were 500 ETB, 4,652.6 ETB and 12,000 ETB, respectively. Eighteen percent of households did not visit health centers to seek treatment for diarrhea. Costs of diagnosis, costs of treatment and drug, and costs of prevention of diarrhea varied largely between households.

3.2. Public health burden of foodborne zoonotic diseases

Based on the number of respondents in each of the towns, the health burden due to foodborne zoonotic diseases in Gondar, Lalibela and Debark towns were 0.2, 0.1 and 1.3 DALYs per household per year, respectively. Respectively, the health burden was estimated at 73.2, 146.6 and 1,689.5 DALYs out of 100,000 populations per year in Gondar, Lalibela and Debark towns. The overall health burden due to foodborne zoonotic diseases (aggregated over the 435 households in the three towns) was estimated to be 89.9 DALYs per 100,000 populations per year (Table 2).

Table 2: Annual health burden due to foodborne zoonotic diseases in disability adjusted life years per 100,000 population in Gondar, Lalibela and Debark towns in Amhara region, Ethiopia.

**Source of health burden**	**Gondar**	**Lalibela**	**Debark**	**Overall**
Years of life lost	38.9	0	1,530.2	66
Disability due to medication	6.5	37.5	23.7	4.6
Disability due to foodborne associated diarrhea	27.8	109.1	135.6	19.3
Total DALY[1]	73.2	146.6	1,689.5	89.9

[1]Disability adjusted life years

3.3. Economic impact of foodborne zoonotic diseases

The economic impact of foodborne zoonotic diseases in the three towns of Amhara regional state, in average, was 279 ETB per household per year. The overall economic burden due to foodborne zoonotic diseases (aggregated over the 435 households in the three towns) was estimated to be 121,355.68 ETB per year. Of the different cost factors, costs of preventive measures' made the highest contribution while costs of diagnosis made the lowest contribution to the total economic impact of foodborne zoonotic diseases (Table 3).

Table 3: Economic impact of foodborne zoonotic diseases on health based on selected households in the community (N= 435).

**Costs**	**Cost factors**	**Gondar**	**Lalibela**	**Debark**	**Total**
Direct Costs	Costs of diagnosis	6,240.64	857.5	1,249.5	8,347.64

	Costs of drugs	6,803.16	1,964.9	3,964.1	12,732.16
Indirect costs	CPWTL[1]	15,000.13	3,356.50	4,814.25	23,170.88
	Costs of informal careers	6,750	2,145	500	9,395
Costs of preventive measures		57,950	2,910	6850	67,710
Total economic impact		92,743.93	11,233.90	17,377.85	121,355.68

[1]Costs of productive working time losses

3.4. Awareness of foodborne zoonotic diseases

From a total of 435 respondents, 305 (70.1%) knew the presence of zoonotic diseases and 275 (90%) of them knew zoonotic diseases can be acquired from food. The awareness that zoonosis can be acquired from food was highest in Debark and it is more in men than in women and this awareness decreased with increased age of respondents. On the other hand the awareness about foodborne zoonotic disease increased with increased the level of education and income. Table 4 summarizes awareness of foodborne zoonosis by different socio-demographic variables.

Table 4: Descriptive statistics showing awareness of foodborne zoonotic diseases by socio-demographic characteristics of study participants in Amhara region, Ethiopia.

Variable	Level	Number of respondents	Aware zoonosis can be acquired from food (%)
Study site	Gondar	286	176 (62)

	Lalibela	71	46 (65)
	Debark	78	53 (68)
Gender	Female	137	80 (58)
	Male	298	195 (65)
Age in years	<35	164	100 (61)
	36-50	201	134 (67)
	>50	70	41 (59)
Level of education	Illiterate	26	8 (31)
	General education	54	24 (44)
	College education	355	243 (68)
Household size	One person	170	87 (51)
	> one person	265	188 (71)
Income category in ETB[1]	< 2500	73	34 (47)
	2501 – 5000	195	111 (57)
	5001- 7500	124	90 (73)
	>7500	43	40 (93)

[1]Ethiopian Birr

3.5. Sociodemographic factors associated with awareness of foodborne zoonotic diseases

Three variables were statistically significant association with awareness of foodborne zoonotic diseases. Table 5 summarizes descriptive statistics on awareness of foodborne zoonotic diseases and univariable associations with socio-demographic characteristics of respondents.

Table 5: Descriptive statistics on awareness of foodborne zoonotic diseases as reflected by respondents and univariable associations with socio-demographic characteristics of 435 respondents in Amhara region, Ethiopia.

**Variable**	**Level**	**OR[1]**	**95% CI[2]**	**P-value**
Study site	Gondar	Ref[3]		
	Lalibela	1.15	(0.67- 1.98)	0.613
	Debark	1.3	(0.78- 2.25)	0.300
Gender	Female	Ref.		
	Male	1.35	(0.89-2.04)	0.158
Age in years	<35	Ref.		
	36-50	1.28	(0.83-1.97)	0.260
	>50	0.90	(0.51-1.60)	0.731
Level of education	Illiterate	Ref.		
	High School	1.8	(0.67-4.85)	0.245
	Diploma and above	4.89	(2.06-11.56)	0.001
Household size	One person	Ref.		
	>one person	2.33	(1.56-3.48)	0.001
Income	< 2500	Ref.		
	2501 – 5000	1.52	(0.88-2.60)	0.131
	5001- 7500	3.04	(1.66-5.57)	0.001

	>7500	15.29	(4.34-53.93)	0.001

[1]Odds ratio

[2]Confidenece interval

[3]Refernce

Level of education, household size and income were statistically significant association with awareness by the multivariable logistic regression model. The final multivariable model is presented in Table 6.

Table 6: The final multivariable mixed models describing the associations between awareness of foodborne zoonotic diseases and respondents socio-demographic characteristics on 435 respondents in Amhara region, Ethiopia.

**Variable**	**Level**	**OR[1]**	**95% CI[2]**	**P-value**
Level of education	Illiterate	Ref[3]		
	High School	2.30	(0.83-6.36)	0.108
	Diploma and above	5.17	(1.86-14.39)	0.002
Household size	One person	Ref.		
	>one person	2.27	(1.48-3.49)	0.001
Income	< 2500	Ref.		
	2501 – 5000	0.76	(0.38-1.530)	0.448
	5001- 7500	1.24	(0.55-2.78)	0.600
	>7500	5.64	(1.43-22.32)	0.014

[1]Odds ratio

[2]Confidenece interval

[3]Refernce

Discussion

The objectives of this study were to estimate impact of foodborne zoonotic diseases on health ~~and~~ economy and to assess demographic factors associated with awareness of foodborne zoonotic diseases in Amhara region, Ethiopia. One of the first prerequisites in making decision to implement measures to control foodborne zoonotic diseases is to perceive foodborne zoonotic diseases ~~are~~ a health burden and are an economic problem. Decision makers, however, do not always perceive foodborne zoonotic diseases as costly, and therefore underestimate these diseases as a problem. Although there are a reasonable number of papers on health burden and economic impact of foodborne zoonotic diseases in the literature, to our knowledge, there is no study so far which estimated the economic and health burden of foodborne zoonotic diseases in Ethiopia. Specifically for developing countries this type of knowledge, although valuable, is lacking. Of the 146 studies recently reviewed for Major Food-Borne Zoonotic Bacterial Pathogens, no study estimated the economic and health burden of foodborne zoonotic diseases [31]. Through estimating economic and health burden of foodborne zoonotic diseases in Ethiopia, the current study shows the need of improving prevention of foodborne zoonotic diseases.

Decision makers are often unaware of the economic impacts of foodborne zoonotic diseases. Creating awareness on the health and economic burden of foodborne zoonotic diseases may be an important step, because people first have to consider foodborne zoonotic diseases as problem before they will be motivated to take measure [42]. This may be helpful in their motivation to

implement preventive measures that (partly) may have been known already, but that were considered to not be outweighing the problem to be solved. Thus giving more insight the health and economic burden associated to foodborne zoonotic diseases may motivate households to change their behavior related to prevention of these diseases.

Collecting data on factors such as additional cost for better food for sick person and better care under the local circumstances was quite challenging. Disability weights for diarrhea and medication associated to foodborne zoonotic diseases were used from the literature. Since respondents didn't know whether a disease affecting one or more of their household members was zoonotic, the response obtained in the questionnaire was extrapolated towards a whole year. Consequently, the number of foodborne zoonotic diseases was obtained based on extrapolating the data obtained in the questionnaire using confirmatory diagnosis from retrospective data in health records.

The economic impact of foodborne zoonotic diseases in three towns of Amhara region, in average, was 279 ETB per household per year. This means that it was equivalent to 0.5% of the average annual income of respondents. Besides the economic impacts described in this paper, it is known that additional costs associated to foodborne zoonotic diseases result from costs related to taxi fee to travel to hospitals seeking health service. However, this cost was not included as both patient and the ~~carer~~ mostly walk to hospitals. This might have underestimated the economic impact of foodborne zoonotic diseases. In absolute terms, the economic impacts of foodborne zoonotic diseases estimated looks small but ~~it significant relative to average income of respondents in Ethiopia~~. So the study indicated that foodborne zoonotic diseases are causing significant economic impact to households in the study area.

There was large variation in the economic impacts of foodborne zoonotic diseases between households. The largest part of the variation can be explained by differences in costs of preventive measures and in costs of treatment and drug. Although we wanted to base the estimate of economic impacts of foodborne zoonotic diseases as much as possible on actual confirmed data, it was not possible to retrieve all needed data from the field. Therefore we had to make a number of assumptions, for instance on costs of labor. Because of the assumption, there may be discrepancies between the real economic impacts of foodborne zoonotic diseases and our estimates. A relatively large part (52%) of the total economic impacts of foodborne zoonotic diseases was due to costs of preventive measures. However, as there is no ~~any~~ previous study, to our knowledge, it was not possible to compare. The contribution of costs of patients' time to the total economic impacts of foodborne zoonotic diseases is caused by the long time taken for treatment, sick leave, and absenteeism from work because of the disease.

Larger majority of respondents (70.1%) had known the presence of zoonotic diseases but only a smaller majority (63.2%) knows disease can be acquired from food. Both findings were substantially lower than the earlier reported in and around Addis Ababa, Ethiopia [26] although the respondent of that study were elementary and high school students, and Graduates of which 33% were health professionals. Level of education, income and household size were associated with awareness of foodborne zoonotic diseases. This may be related to the fact that more level of education has direct relevance to awareness related health and diseases. Households with more than one person in a house had higher odds of awareness of foodborne zoonotic diseases than households of one person in a house. This one is explained from the fact that if ~~the~~ more people

live in the household, there is more chance that at least one of them will be aware and transmits this awareness to other members of the household.

This study aimed to assess the burden of foodborne zoonotic diseases in Amhara region, Ethiopia in terms of DALYs. The estimates might not have been very accurate as cases were determined by extrapolation of few confirmed cases based on retrospective data. Moreover, we found that not all cases with diarrhea seek health service. As acknowledged by WHO [43], the high life expectancy allocated and the relatively higher number of cases and death to children result in higher DALYs of foodborne zoonotic diseases in these age groups. Extrapolation to the national scale can provide an indication of the health and economic burden associated to foodborne zoonotic diseases in the country. However, the estimate should be interpreted with caution, given the relative limited number of towns studied and the nature of the data.

Conclusions

Foodborne zoonotic diseases were associated with high health and economic burden in Amhara Region, Ethiopia. The overall health burden due to foodborne zoonotic diseases (aggregated over the 435 households in Gondar, Lalibela and Debark towns) was estimated to be 89.9 DALYs per 100,000 populations per year. On average, the economic burden was 278.98 ETB per household per year and show large variation between households. Relative to the income of households, these costs are high. The total costs of foodborne zoonotic diseases were mainly due to costs of preventive measures. Although further study is needed at some points, respondents socio-demographic characteristics associated with awareness of foodborne zoonotic diseases were identified, that can be helpful in tailoring in control of foodborne zoonotic diseases in Amhara region, Ethiopia.

Supporting information

S1

(DOCX)

S2 Table

(CSV)

Acknowledgements

We gratefully acknowledge respondents who participated in the study. We are also thankful to medical directors who allow us to collect retrospective data from health records.

References

1. Woolhouse ME, Gowtage-Sequeria S. Host range and emerging and reemerging pathogens. Emerg Infect Dis. 2005; 11:1842. https://doi.org/10.3201/eid1112.050997.

2. Jones KE, Patel NG, Levy MA, Storeygard A, Balk D, Gittleman JL, et al. Global trends in emerging infectious diseases. Nat Lett. 2008 451:990–994. https://doi: 10.1038/nature06536.

3. The control of neglected zoonotic diseases-a route to poverty alleviation. Report of a joint WHO/DFID-Animal Health Programme meeting with the participation of FAO and OIE, World Health Organization, Geneva, Switzerland. 2006.

4. Pieracci GE, Hall JA, Gharpure R, Haile A, Walelign E, Deressa A, Bahiru G, et al. Prioritizing zoonotic diseases in Ethiopia using a one health approach. One Health. 2016; 2: 131–135. http://dx.doi.org/10.1016/j.onehlt.2016.09.001.

5. People, Pathogens and Our Plant, Vol 1: Towards a Once Health Approach for Controlling. Zoonotic Diseases Report 50833-GLB, World Bank. 2010.

6. Grace D, Gilbert J, Randolph T, Kang'ethe E. The multiple burdens of zoonotic disease and an ecohealth approach to their assessment. Trop Anim Health Prod. 2012; 44: 67–73. http://doi 10.1007/s11250-012-0209-y.

7. Molyneux D, Hallaj Z, Keusch GT, McManus DP, Ngowi H, Cleaveland S, et al. Zoonoses and marginalised infectious diseases of poverty: where do we stand? Parasit Vectors. BioMed Central Ltd. 2011; 4:106. http://doi:10.1186/1756-3305-4-106.

8. Seimenis A. Zoonoses and poverty - a long road to the alleviation of suffering. Vet Ital. 2012; 48: 5–13.

9. Cleaveland S, Sharp J, Abela-Ridder B, Allan KJ, Buza J, Crump JA, et al. One Health contributions towards more effective and equitable approaches to health in low- and middle-income countries. Phil Trans R Soc B. 2017: 372: 20160168. http://dx.doi.org/10.1098/rstb.2016.0168.

10. Greger M. The human/animal interface: Emergence and resurgence of zoonotic infectious diseases. Crit Rev Microbiol. 2007; 33: 243–299. http://doi: 10.1080/10408410701647594.

11. Cascio A, Bosilkovski M, Rodriguez-Morales JA, Pappas G. The socio-ecology of zoonotic infections. Clin Microbiol Infect. 2011; 17: 336–42. http://doi: 10.1111/j.1469-0691.2010.03451.x.

12. Narrod C, Zinsstag J, Tiongco M. A One Health Framework for Estimating the Economic Costs of Zoonotic Diseases on Society. EcoHealth. 2012; 9: 150–162. http://doi: 10.1007/s10393-012-0747-9.

13. Mapping of Poverty and Likely Zoonoses Hotspots, Zoonoses Project 4, Report to Department for International Development, UK. International research Institute, July 2012.

14. Victoria NG, Sargeant JM. A quantitative approach to the prioritization of zoonotic diseases in North America: a health professionals' perspective. PLoS One. 2013; 8: e72172. https://doi.org/10.1371/ journal.pone.0072172 PMID: 23991057.

15. Leta S, Mesele F. Spatial analysis of cattle and shoat population in Ethiopia: growth trend, distribution and markets access, Singerplus. 2014; 3: 310. https://doi:10.1186/2193-1801-3-310.

16. Menghistu HT, Hailu KT, Shumye NA, Redda YT. Mapping the epidemiological distribution and incidence of major zoonotic diseases in South Tigray, North Wollo and Ab'ala (Afar), Ethiopia. Plos One. 2018; 13: e0209974. https://doi.org/10.1371/journal.pone.0209974.

17. Tadesse G, Tessema T. A meta-analysis of the prevalence of Salmonella in food animals in Ethiopia. BMC Microbiology. 2014; 14: 270. https://doi:10.1186/s12866-014-0270-y.

18. Hoelzer K, Switt AIM, Wiedmann M. Animal contact as a source of human non-typhoidal salmonellosis. Veterinary Research. 2011; 42: 1–27. https://doi:10.1186/1297-9716-42-34.

19. Dargatz DA, Fedorka-Cray PJ, Ladely CA, Kopral CA, Ferris KE, Headrick ML. Prevalence and antimicrobial susceptibility of Salmonella spp. isolates from US cattle in feedlots in 1999 and 2000. Journal of Applied Microbiology. 2003; 95:753–761. https://doi:10.1046/j.1365-2672.2003.02034.x.

20. Molla B, Roman Y, Daniel A. *Listeria monocytogenes* and other *Listeria* species in retail meat and milk products in Addis Ababa, Ethiopia. Ethiop J Health Dev. 2004; 18: 1–5. https://doi: 10.4314/ejhd.v18i3.9962.

21. Meloni D, Galluzzo P, Mureddu A, Piras F, Griffiths M, Mazzette R. *Listeria monocytogenes* in RTE foods marketed in Italy: prevalence and automated EcoRI ribotyping of the isolates. Int J Food Microbiol. 2009; 129: 166-173. https://doi: 10.1016/j.ijfoodmicro.2008.11.014.

22. Mengesha D, Zewde BM, Toquin MT, Kleer J, Hildebrandt G, Gebreyes WA. Occurrence and distribution of *Listeria monocytogenes* and other Listeria species in ready-to-eat and raw meat products. Berl Munch Tierarztl Wochenschr. 2009; 122: 20-24.

23. Craig PS, McManus DP, Lightwolers MW, Chabalgoity JA, Garcia HH, Gavidia CM, et al. Prevention and control of cystic Echinococcosis. Latent Infec Dis. 2007; 7: 38-94. https://doi: 10.1016/S1473-3099(07)70134-2.

24. Shitaye J, Getahun B, Alemayehu T, Skoric M, Treml F, Fictum P, et al. A prevalence study of bovine tuberculosis by using abattoir meat inspection and tuberculin skin testing data, histopathological and IS6110 PCR examination of tissues with tuberculous lesions in cattle in Ethiopia. Veterinarni Medicina-Praha. 2006; 51: 512.

25. Tassew H, Abdissa A, Beyene G, Gebre-Selassie S. Microbial flora and food borne pathogens on minced meat and their susceptibility to antimicrobial agents. Ethiopian Journal of Health Sciences. 2011; 20: 137–143. https://doi: 10.4314/ejhs.v20i3.69442.

26. Kumar A, Tadesse G. Bovine Cysticercosis in Ethiopia: a review. Ethiop Vet J. 2011; 15:15–35. https://doi: 10.4314/evj.v15i1.67681.

27. Girma S, Zewde G, Tafess K, Jibat T. Assessment of awareness on food borne zoonosis and its relation with Veterinary Public Health Services in and around Addis Ababa, Ethiopia. Ethiop Vet J. 2012; 16: 15-22. https://doi: 10.4314/evj.v16i1.2.

28. Aklilu A, Kahase D, Dessalegn M, Tarekegn N, Gebremichael S, Zenebe S, et al. Prevalence of intestinal parasites, Salmonella and Shigella among apparently health food handlers of addis ababa university student's cafeteria, Addis Ababa, Ethiopia. BMC Research Notes. 2015; 8:17. https://doi.org/10.1186/s13104-014-0967-x.

29. Tadesse G, Gebremedhin ZE. Prevalence of Salmonella in raw animal products in Ethiopia: a meta-analysis. BMC Research Notes. 2015; 8: 1–8. https://doi 10.1186/s13104-015-1127-7.

30. Kemunto N, Mogoa E, Osoro E, Bitek A, Njenga MK, Thumbi SM. Zoonotic disease research in East Africa. BMC Infectious Diseases. 2018; 18: 545. https://doi.org/10.1186/s12879-018-3443-8.

31. Abebe E, Gugsa G, Ahmed M. Review on Major Food-Borne Zoonotic Bacterial Pathogens. Journal of Tropical Medicine. 2020; 4674235. https://doi.org/10.1155/2020/4674235.

32. Population projection of Ethiopia for all regions at Wereda level from 2014–2017.CSA. Central Statistical Agency (CSA), Federal Democratic Republic of Ethiopia, Addis Ababa. 2013.

33. Seleshe S, Jo C, Lee M. Meat Consumption Culture in Ethiopia. Korean J. Food Sci. An. 2014; 34: 7-13. https://doi: 10.5851/kosfa.2014.34.1.7.

34. Potts SK, Mulugeta A, Bazzano AN. Animal Source Food Consumption in Young Children from Four Regions of Ethiopia: Association with Religion, Livelihood, and Participation in the Productive Safety Net Program. Nutrients. 2019; 11: 354. https://doi: 10.3390/nu11020354.

35. Abegaz GA, Hassen HI, Minten B. Consumption of Animal-source Foods in Ethiopia: Patterns, Changes, and Determinants. Working paper, 113, Ethiopian Development Research Institute (EDRI), Strategy support program. 2018.

36. Human population census of Ethiopia. Central Statistical Agency (CSA), Federal Democratic Republic of Ethiopia, Addis Ababa. 1999.

37. Havelaar AH, Kirk MD, Torgerson PR, Gibb HJ, Hald T, Lake RJ, et al. World Health Organization Global Estimates and Regional Comparisons of the Burden of Foodborne

Disease in 2010, PLoS Med. 2015; 12: e1001923. https://doi.org/10.1371/journal.pmed.1001923.

38. Torgersona RP, Rüegga S, Devleesschauwerb B, Abela-Ridderd B, Havelaare AH, Shawg APM, et al. An adjusted indicator to estimate the burden of zoonotic diseases. One health. 2018; 5: 40-45. https://doi: 10.1016/j.onehlt.2017.11.003.

39. Salomon AJ, Haagsma AJ, Davis A, de Noordhout MC, Polinder S, Havelaar HA. Disability weights for the Global Burden of Disease 2013 study. Lancet Glob Health. 2015; 3: e712–23. https://doi. 10.1016/S2214-109X(15)00069-8.

40. Haagsma JA, de Noordhout CM, Polinder S, Vos T, Havelaar AH, Cassini A. Salomon. Assessing disability weights based on the responses of 30,660 people from four European countries. Popul Health Metr. 2015; 13: 10. https://doi: 10.1186/s12963-015-0042-4.

41. McIntyre D, Thiede M, Dahlgren G, Whitehead M. What are the economic consequences for households of illness and of paying for health care in low- and middle-income country contexts? Social Science and Medicine. 2006; 62: 858-865. https://doi: 10.1016/j.socscimed.2005.07.001.

42. Janz NK, Becker MH. The health belief model: A decade later. Health Education Quarterly. 1984; 11: 1–47. https://doi: 10.1177/109019818401100101.

43. WHO methods and data sources for global causes of death, 2000-2015. Global Health Estimates Technical Paper WHO, World Health Organization, Geneva. 2017.

Click here to access/download
Supporting Information
Supplementary material 1.doc

Click here to access/download
Supporting Information
CSV.csv
