# Supplementary material for: Health and economic burden of foodborne zoonotic diseases in Amhara region, Ethiopia
Source: PLoS One. 2021 Dec 31;16(12):e0262032. doi: 10.1371/journal.pone.0262032 (PMC8719781; doi:10.1371/journal.pone.0262032)
Supplement: Attachment — Submitted filename: Response to Reviewers.doc [file pone.0262032.s004.doc]

**Response to the editor and reviewers’ comments**

When submitting your revision, we need you to address these additional requirements.

1. Please ensure that your manuscript meets PLOS ONE's style requirements, including those for file naming. The PLOS ONE style templates can be found at

***We appreciate this thorough review and proper feedback. Indeed, we agree with this reviewer that there are several flaws and we have corrected according to the reviewers’ comments.***

***We checked the STROBE guidelines and mentioned the study design in the abstract.***

***We highlighted all changes in yellow in the original version of the manuscript and we responded to all specific comments below, indicating line numbers in the revised manuscript where changes were made.***

2. We note that the grant information you provided in the ‘Funding Information’ and ‘Financial Disclosure’ sections do not match.

***We appreciate pointing this out. Now, we made the grant information in the ‘Funding Information’ and ‘Financial Disclosure’ sections to mach and provided grant numbers for the awards we received in the ‘Funding Information’ section.***

Additional Editor Comments (if provided):

This manuscript investigates health and economic burden of foodborne zoonotic diseases in Amhara region, Ethiopia. I commend the authors for conceptualizing this study, as the findings certainly add value to the literature. I have following comments:

1) The title of the manuscript should be framed as per STROBE guideline.

***We thank the editor. Now, the study’s design was mentioned in the abstract; line 11.***

2) Psychometrics of the survey instruments should be provided.

***To check validity of the questionnaire, the original English questionnaire was translated into local language (Amharic), and subsequently translated back into English by an external translator to validate the translation. Then the questionnaire was pilot-tested by a administering to 5 people that have similar characteristics. Finally, the questionnaire was corrected based on the response of the pilot test before the final questionnaire. Now, clear description is given in lines 148-152.***

3) Sample size calculations including power analysis should be provided

***We agree with the reviewer’s comment. When the sample size was set at 435 households, we used our own expertise estimate and the balance of local logistic possibilities, data collecting costs (time and money) and enrolling representative numbers of samples of the population of households. However, this sample size is more than the sample size that can be calculated at 80% power at 95% confidence interval.***

4) Authors said they used multiple logistic regression model. However, there is a lack of clarity on what basis the model was built. Whether they used a-priori hypothesis or any stepwise input of parameters. This information should be provided in data analysis section.

***We agree that this was unclear. We now explained this in lines 290-292.***

5) Table#5: please correct title of the table as per the data.

***We made correction on the title of Table 5 based on the data contained in the table (Lines 368-370).***

Reviewer #1

**Material and methods**:

• What is meant by projected population?

***Since population census was not made recently, we used the population projection figures made by the Central Statistical Authority of Ethiopia. That population projection was made based on the results of National population and Housing Census of Ethiopia conducted in May 2007. Now it made clear in lines 108-109.***

• Sample size and sampling method not properly documented. How 435 households were selected from 3 towns and method of recruitment of the respondents in the study is not clear.

***We appreciate the reviewer points this out this flaw in our methodology. We rewrote this section aiming to more clearly express the sample size and sampling method. Changes were made in lines 113 to 119.***

• The term “haphazardly recruited” is not statistically correct.

***The reviewer is correct that it was not statistically correct. Now, correction has been made in lines in 120-122.***

• Animal source food consumption habits should be documented in either introduction or results or discussion.

***Thank you for the reviewer for pointing this out. Animal source food consumption habits are explained in the introduction section in lines 77 to 81.***

• Grammatical errors are there in the text. It should be written in past tense but at some places, it is written in present or future tense.

***We agree with the reviewer’s comment. We read the manuscript and made a number of corrections such as in lines: 390, 404, and 450-451.***

• In data management and data analysis, write down the methods/software used e.g. Excel, SPSS etc.

***The software used is explained in lines 297 to 298.***

**Results**
• Did not discuss the socio-demographic profile of the respondents in the starting of the results.

***The reviewer was correct that discussing socio-demographic profile of respondents will add information. Now, socio-demographic profile of the respondents is added at the start of the results in lines 301-305.***

• Have not mentioned the risk factors or food consumption habits here.

***Possible risk factors or food consumption habits are explained in lines 135-140.***

**Discussion**
• Comparison with similar earlier studies is not sufficient.

***The reviewer is correct. When we search and read papers estimated the economic and health burden of foodborne zoonotic diseases, none of them referring about developing country. Now, this clearly described in lines 396-398. Therefore, it was not possible to made comparisons with such reports as they are in different set up such as different level of economic background and level of knowledge.***

Overall remarks

• The study findings are good in terms of health and economic burden. But need to rectify the material and methods. Also there is need to pay focus on risk factors and food consumption habits. Also give recommendations based on the findings of the study.

***We appreciate the reviewer points out in our methodology. Now, the sample size, sampling methods, and animal source food consumption habits and risk factors are described in the methods section in lines 113-140. Recommendations are given based on the findings of the study in lines 473-474.***

Reviewer #2:

 line 31- delet is and include 'may be'

***We have made the correction: ‘is’ is deleted and ‘may be’ is included.***

line - 97- insert 'literature' after the word no

***Corrected based on the reviewer’s comment, line 98.***

I had made corrections in the sticky notes and uploaded the pdf paper.

***We thank the reviewer for giving corrections in sticky notes in the pdf of the original manuscript. All corrections in the sticky notes are included in respective places in the revised manuscript.***
